# A multifunctional drug consisting of tetracycline conjugated with odanacatib for efficient periodontitis therapy

**DOI:** 10.3389/fphar.2022.1046451

**Published:** 2022-10-26

**Authors:** Dengke Li, Wuyang Zhang, Weiliang Ye, Yuan Liu, Yuan Li, Yiming Wang, Bingqing Shi, Xueni Zheng, Ying An, Zhen Ma, Kaijin Hu, Hongzhi Zhou, Yang Xue

**Affiliations:** ^1^ State Key Laboratory of Military Stomatology & National Clinical Research Center for Oral Diseases & Shaanxi Clinical Research Center for Oral Diseases, Department of Oral and Maxillofacial Surgery, School of Stomatology, The Fourth Military Medical University, Xi’an, Shaanxi, China; ^2^Department of Stomatology, Air Force Hospital of Southern Theater Command, Guangzhou, Guangdong, China; ^3^ Department of Pharmaceutics, School of Pharmacy, The Fourth Military Medical University, Xi’an, Shaanxi, China; ^4^ Department of Oral Histology and Pathology, School of Stomatology, The Fourth Military Medical University, Xi’an, Shaanxi, China; ^5^ Department of Periodontology, School of Stomatology, The Fourth Military Medical University, Xi’an, Shaanxi, China

**Keywords:** multifunctional drug, cathepsin K, odanacatib, periodontitis, osteoporosis

## Abstract

The treatment of periodontitis can be very challenging due to its complex etiologies. A new pharmacologic strategy entitled “host-modulation therapy,” has been introduced to improve periodontal treatment outcomes. Supposedly, a multifunctional drug with the potential for bacterial infection prevention, host-response modulation and bone healing promotion would be a promising option for periodontitis therapy, but related studies remain substantially lacking. In this study, we successfully conjugated tetracycline with odanacatib (a selective inhibitor of cathepsin K) to construct a multifunctional drug (TC-ODN). We discovered that TC-ODN could promote macrophages polarizing toward anti-inflammatory phenotype and promote osteogenesis of PDLSCs under inflammatory microenvironment. *In vivo*, TC-ODN could be absorbed and distributed specifically to the bone after systemic administration, and accumulation of TC-ODN increased bone mineral density in ovariectomized rats. Importantly, periodontal administration of TC-ODN could successfully promote bone healing in periodontitis rats with alveolar bone loss. The findings in our study uncovered the excellent biocompatibility and multifunction of TC-ODN, including bone-targeted accumulation, immunoregulation, anti-inflammatory activity and promotion of bone healing, which might contribute to the clinical treatment of periodontitis.

## Introduction

Periodontitis is a bacterial infection disease resulting in local inflammation and osteoclastic damage of the alveolar bone, which ultimately leads to tooth loss (Slots, 2000; Tsukasaki, 2021). Up to 5%–25% adults around the world is suffering from severe periodontitis, making this disease the sixth most prevalent human disease (Sanz et al., 2020; Wei et al., 2021). Due to the complex etiologic factors, the traditional therapeutic strategy by using systemic antimicrobial agents adjunctive to mechanical/surgical periodontal treatment can hardly provide curative effects for patients with aggressive or severe periodontitis (Slots, 2000; Wei et al., 2021). Any factor that affects either the periodontal environment or the host response may contribute to the progression of the disease and poor treatment response. To improve the efficacy and cost-effectiveness of periodontal therapy, innovative pharmacologic strategies are needed (Slots, 2000; Wei et al., 2021). Theoretically, a multifunctional drug, which exhibits antimicrobial, anti-inflammatory, and bone healing promotive properties, would be a promising option in the management of periodontitis, but related studies remain substantially lacking.

Tetracycline is one of the most extensively used drugs in treating refractory periodontal disease because of its antibiotic properties against both gram-positive and gram-negative bacteria. Particularly, tetracycline can concentrate in the periodontal tissues and inhibit the growth of A. actinomycetemcomitans, which is associated with localized aggressive periodontitis or severe periodontitis (Prakasam et al., 2012). Moreover, it has been highlighted in recent years that tetracycline also exerts host regulatory actions besides their anti-microbial activity, including anti-inflammatory activities and inhibition of matrix metalloproteinases (MMPs) and osteoclast activities (Vernillo et al., 1994). MMPs and other osteoclast-derived enzymes are major drivers of the aggressive destruction of alveolar bone and supporting connective tissues (Giannobile, 2008). Therapeutic approaches targeting MMPs inhibition permit the control of periodontitis in high-risk patients susceptible to disease reactivation or disease complications. Chemically modified tetracyclines have been widely investigated as an important MMPs inhibitor (Giannobile, 2008). However, the impact of tetracyclines on physiologic bone remodeling or bone regeneration is unclear. The effects of tetracycline antibiotics on osteoblasts have drawn far less attention than on osteoclasts. A literature review has shown that tetracycline antibiotics have divergent dose-dependent effects on osteoblastogenesis (Warner et al., 2022). In detail, a higher dose of tetracycline may inhibit osteoblast function and proliferation. There is no report of effective treatment by using tetracycline alone to repair inflammatory bone loss in periodontitis.

The osteoclast resorbs bone by secretion of proteolytic enzymes that primarily belong to two families of proteinases: the cysteine proteinases and the MMPs (Delaisse et al., 2003; Everts et al., 2006). The cysteine proteinase, cathepsin K(CTSK), is highly expressed in osteoclasts and taken to be essential in bone resorption (Troen, 2004; Beklen et al., 2015; Wen et al., 2016). As we all know, MMPs only participate in bone degradation. In our previous study, we demonstrated for the first time that the deficiency of CTSK can promote alveolar bone regeneration in mouse tooth extraction models (Zhang et al., 2021). We further proved that inhibition of CTSK promotes jaw bone marrow mesenchymal stem cell proliferation and osteoblast differentiation. Recent studies also have shown that deletion of CTSK can regulate the host immune response to periodontal pathogens and prevent local inflammation by significantly decreasing the number of immune cells (T cells and macrophages) (Hao et al., 2015a; Chen et al., 2016). Inhibition of CTSK can generate a viable local immune microenvironment that promotes cell homing and tissue formation, thereby achieving higher levels of tissue repair (Yang et al., 2021). Based on our findings and the analysis above, we hypothesized that conjunctive application of MMPs inhibitor (tetracycline) and CTSK inhibitor could additively maintain a protective state for periodontal tissue, and potentially enhance bone repair in periodontal diseases.

Notably, it was found that the activity of CTSK has far-reaching effects throughout various organs besides bone (Dai et al., 2020). CTSK has been found widely distributed in the central nervous system, cardiovascular system, respiratory system, and other organs and systems. CTSK inhibition in non-bone sites may cause undesirable adverse effects. Odanacatib developed by Merck & Co. is the only small molecular CTSK inhibitor candidate which demonstrated high therapeutic efficacy to increase bone mineral density in patients with osteoporosis (McClung et al., 2019; Stone et al., 2019). What’s more, the preventive effect of odanacatib has also been explored in a bacteria-induced periodontitis mouse model and confirmed by preventing bone loss and the immune response during the progression of periodontitis (Hao et al., 2015b). However, odanacatib was stagnated due to the risk of stroke, and there is no report investigating how to avoid side effects of CTSK inhibitor in non-bone organs (Mullard, 2016). Tetracycline has the well-known characteristics of bone targeting. Not only newly-formed bone but also mineralized dead bone can take up tetracyclines on their surface. To the best of our knowledge, this is the first attempt to construct a bone-targeted CTSK inhibitor *via* conjugation of odanacatib with tetracycline.

## Manuscript formatting

### Methods

#### Fabrication and characterization of TC-ODN

Odanacatib-oxalyl chloride conjugates were synthesized by the esterification reaction between amino group of odanacatib (MedChemExpress, China) and chlorine group of oxalyl chloride. Next, TC-ODN conjugates were synthesized by the esterification reaction between hydroxy group of tetracycline and chlorine group of odanacatib-oxalyl chloride. A nuclear magnetic resonance spectrometer (BioSpin AC-80; Bruker Optik GmbH, Ettlingen, Germany) was used to obtain the ^1^H-NMR spectra of the products. In addition, mass spectrometry (MS) of the products was obtained using Waters Quattro Premier LC−MS/MS system (Milford, MA, United States) and MassLynx 4.1 software.

#### Preparation of fluorescein

Odanacatib-oxalyl chloride (or TC-ODN) and FITC were dissolved in dimethyl sulfoxide and stirred at room temperature. Then, the product was dialyzed through a dialysis membrane (MW cut-off, 500Da; Solarbio, China) for 48 h. The final product was lyophilized and stored at 4°C, protected from light for further use.

#### Cell culture

For the culture of periodontal ligament stem cells (PDLSCs), The first and second molars of eight-week-old male Sprague-Dawley (SD) rats were extracted, and periodontal ligaments were collected from the middle segment of the root. The tissue was digested with type I collagenase (2 mg/ml) and dispase II (4 mg/ml) at 37°C for 1 h. After passing through a 70 μm strainer, single-cell suspensions were cultured in DMEM (high glucose) supplemented with 10% fetal bovine serum (FBS) and 1% penicillin/streptomycin (Hyclone, United States). PDLSCs at passage three were used for the following experiments.

For the culture of macrophages, the femurs and tibias of three-week-old SD rats were harvested and the bone marrow was rinsed, followed by erythrocyte lysis by red blood cell lysis buffer (Solarbio, China). The cells were resuspended. After 24 h of incubation, the supernatant was collected and centrifugated. The cells were resuspended in media with M-CSF (30 ng/ml) and sustained for 7 days to induce the maturation of macrophages.

All SD rats were purchased from the Animal Center of the Fourth Military Medical University, Xi’an, China. All animal experiments were performed in strict accordance with protocols approved by the Animal Care Committee of the Fourth Military Medical University, China (Approval ID 2019-022).

#### Cellular uptake, cell toxicity, and proliferation assay

The cell toxicity and proliferation assay were detected by Cell Counting Kit-8 (CCK-8, Solarbio, China) according to the manufacturer’s instructions. PDLSCs were incubated with a medium containing TC-ODN-FITC or odanacatib-FITC (1 μmol/L). At a pre-determined time, cells were fixed and permeated, followed by incubated with phalloidin. The nucleus was counterstained with DAPI. Fluorescence imaging was performed under confocal microscopy.

#### Gelatin zymography assay

Pro-cathepsin K (4.5 μg) was mixed with 5×protein loading buffer without boiling. The mixture was separated and washed in a 2.5% Triton X-100 solution. Then, the gel was incubated with a solution (50 mmol/L Tris-HCl (pH 7.4), 2.5 mmol/L DTT, 2.5 mmol/L EDTA) for 48 h at 37°C. After incubation, Coomassie brilliant blue R-250 was used for gel staining, and then the gel was de-stained in a buffer (10% acetic acid and 30% methanol, diluted with double-distilled water) until proteolytic bands were visualized. The bands were quantitatively analyzed with ImageJ software (National Institutes of Health).

### Western blot, reverse transcription-quantitative polymerase chain reaction, and immunofluorescence staining

The cells were lysed by RIPA buffer (Solarbio, China) for 30 min and then centrifugated. The proteins were quantified by a BCA protein assay kit (Proandy, China). The protein samples were loaded into the Bio-Rad Electrophoresis System and transferred. After blocking in 5% skim milk (Solarbio, China), primary antibodies, β-actin (Abcam, United States), CTSK (Abcam, United States), inducible nitric oxide synthase (iNOS, Abcam, United States), CD206 (Abcam, United States), arginase-1 (Arg1, Proteintech, United States), Toll-like receptor-4 (TLR-4, Abcam, United States), I-kappa-B-α (IKBα, Abcam, United States), IKBα (phospho S36), RUNX2 (Abcam, United States), and Osterix (Abcam, United States) were incubated overnight. Then, the corresponding secondary antibodies were incubated for 1 h. The membranes were developed using ECL Western Blot Substrate Kit (4A Biotech, China) and evaluated with an imaging system (Bio-Rad, United States).

Total RNA was extracted by RNAiso reagent (TaKaRa, Japan). After verification of the purity and quantification, 500 ng of RNA was reverse-transcribed into complementary DNA (cDNA) by using a PrimeScript RT Master Mix (TaKaRa, Japan). And then, RT-PCR was performed with the TB Green Premix EX Taq Ⅱ (TaKaRa, Japan). Primer sequences for Actin, iNOS, and CD206 were shown in Supplementary Table S1. The reaction was carried out according to the suggestions in ABI 7500 real-time PCR system (ABI 7500, United States).

For the immunofluorescence staining, the cells were blocked with BSA (37°C, 1 h) and incubated overnight with CD206, iNOS, CTSK at 4°C. After that, the cells were incubated with the corresponding secondary antibodies followed by counterstaining with DAPI for 5 min. Fluorescence imaging was performed by confocal microscopy.

#### Alkaline phosphatase activity assay and alizarin red staining

ALP activity assay (Beyotime, China) and Alizarin red S (Solarbio, China) were performed according to the manufacturer’s instructions. And the image was performed using a microscope imaging system (Olympus, Japan), and the quantitation of alizarin red staining was performed using 10% cetylpyridine chloride for 30 min and absorbance of each well at 562 nm was recorded.

#### Wound healing test

The cells were scraped by a sterilized 200-μL pipette tip. The FBS concentration was reduced to 1%. The image was obtained at 0 h and 48 h using a microscope imaging system. Quantitation was performed by measuring the gap area using ImageJ software.

#### The bone-targeted capacity of TC-ODN

SD rats were injected with TC-ODN-FITC or odanacatib-FITC (4.75 mmol/kg) *via* tail vein injection. At 3, 6, 12, 24, and 48 h after administration, the organs (brain, heart, lung, liver, spleen, kidney, bilateral femur/tibia, and bilateral maxilla/mandible) were collected. The fluorescence image was performed using an IVIS imaging system (IVIS Lumina X5, PerkinElmer, United States), and the fluorescence signal in those organs was analyzed for quantitative study by LivingImage software (PerkinElmer, United States).

#### Micro-computed tomography and biomechanical assay

To evaluate the effectiveness of TC-ODN on osteoporosis, not only the histomorphological parameters of the femur but also the biomechanical characteristics of the femur and the microscopic biomechanical characteristics of the jaw were examined. Twelve healthy eight-week-old female SD rats underwenteither ovariectomy (OVX, *n* = 9) or sham operation (Sham, *n* = 3). After anesthetized by 2% pentobarbital sodium (0.25 ml/100 g rat, i.p.), the surgery was performed through a ventral abdominal transverse incision. Osteoporosis was induced by bilaterally ovariectomized surgery. In sham operation, only a piece of adipose tissue near the ovary were removed. After 8 weeks of induction, all the OVX rats were divided into three groups randomly as follows: carboxymethyl cellulose (CMC) group, odanacatib group (4.75 mmol/kg/week), TC-ODN group (4.75 mmol/kg/week). The sham group was treated with CMC. All rats were orally administered once a day and were sacrificed at 8 weeks after the first treatment. After treatment, the femurs and mandibles were collected. The distal femurs were scanned with a Micro-CT scanner (78 kV, 100 μA, 22.499 μm, Yokohama, Japan). A volume of interest (VOI) starting at 1 mm below the growth plate, and the morphological parameters of trabecular bone microarchitecture were measured. For the three-point bending test, the femur was fixed with a distance of 20 mm at both ends and compressed under load at a crosshead speed of 2 mm/min until destruction using a bone material testing machine (MZ-500D, Tokyo, Japan). For the nanoindentation test, 4 mm-thick mandibular cortical bone blocks were obtained from the anterior segment of the alveolar ridge. Samples were inlaid with self-curing resin and the surface was polished using waterproof paper (800, 1,000, 1,500, 2,000, 3,000 grits). Then the test was performed using a nanomechanical testing instrument (Bruker Hysitron TI980, United States). The experimental design of this study is illustrated in Figure 5A.

#### Establishment of a rat periodontitis model

To evaluate the effects of TC-ODN in periodontitis, a rat experimental periodontitis model was adopted in this section. After anesthetized, the maxillary first molar was gently tied by 3–0 silk ligature which was infiltrated by lipopolysaccharides (LPS) and maintained for 4 weeks. After removing the ligature, saline, odanacatib, or TC-ODN (10 μL, 1 μmol/L, every other day for 8 weeks) was locally injected into the periodontal pocket. After treatment, the maxilla was collected and scanned. The linear distance from the cementoenamel junction to the alveolar bone crest of the distal palatal root of the maxillary first molar was measured to determine alveolar bone height in periodontitis rats. The experimental design of this study is illustrated in Figure 6A.

#### Histology and immunohistochemistry staining

The sections were stained with hematoxylin & eosin (H&E) staining and tartrate-resistant acid phosphatase (TRAP) staining (Sigma, United States). To investigate the difference in osteogenesis, the sections were incubated with hydrogen peroxide and antigen retrieval. Sections were incubated with 5% BSA solution (Boster, China), and then incubated with primary antibodies against Osterix (1:200, Abcam, United States) at 4°C overnight. Then, sections were incubated with the corresponding secondary antibodies for 1 h. The immunoreactive cells were visualized through the addition of a DAB peroxidase substrate kit (Boster, China) and counterstained with hematoxylin. Osterix-positive cells were counted.

#### Statistical analysis

Data are presented as means ± SD of at least triplicate measurements. Statistical analysis was performed using GraphPad Prism Software (San Diego, United States). Comparisons between two groups were analyzed by a two-tailed, unpaired Student’s t-test. Comparisons between more than two groups were performed by one-way ANOVA. Statistical significance was defined as: *p < 0.05, **p < 0.01, ***p < 0.001.

## Results

### Fabrication and characterization of a multifunctional drug (TC-ODN)

**FIGURE 1:**
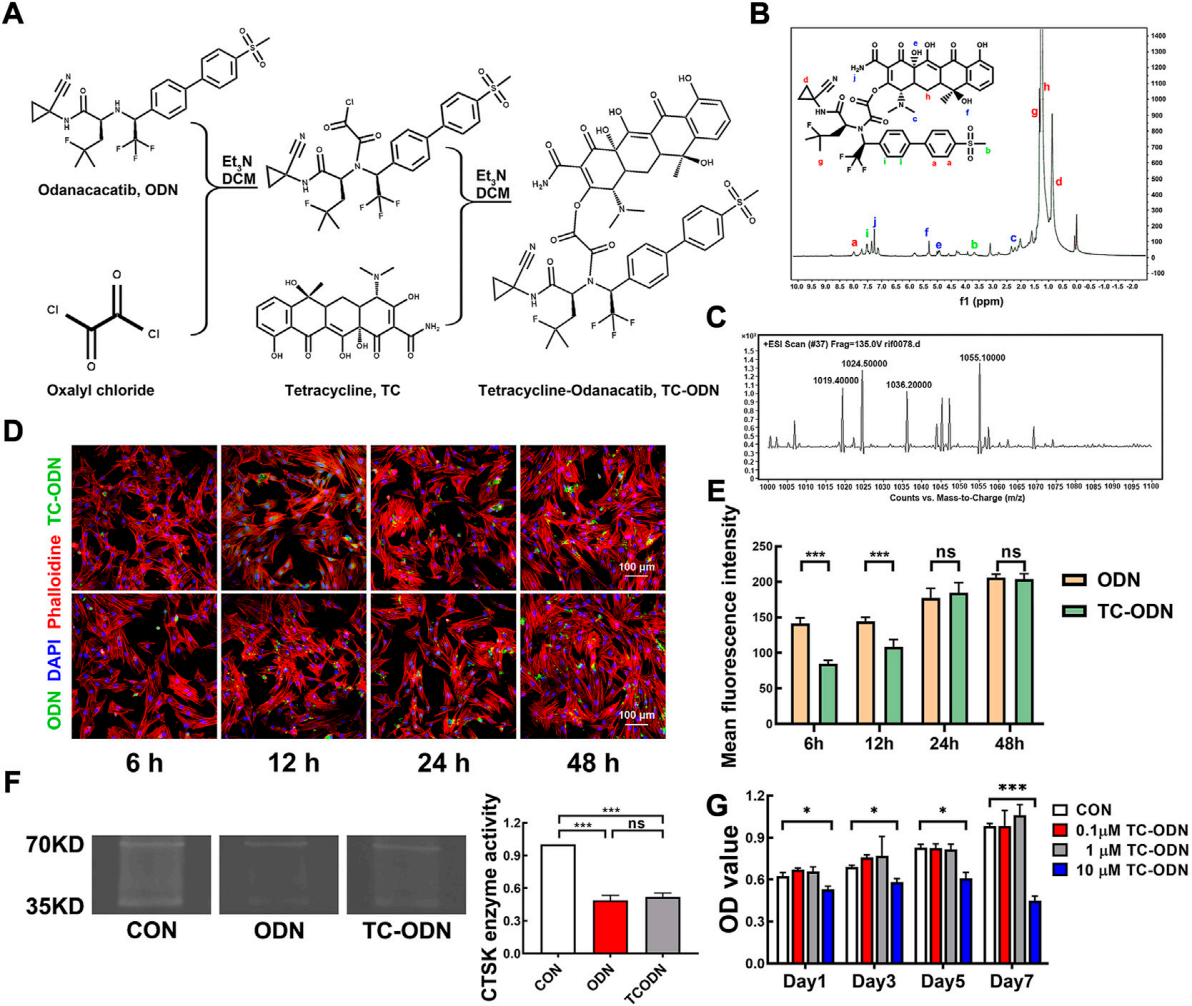
Fabrication and characterization of multifunctional drug (TC-ODN). **(A)** Indirect conjugation of the amino group of odanacatib with the hydroxyl group of TC *via* oxalyl chloride. **(B)** ^1^H-NMR spectroscopy of TC-ODN. **(C)** Mass spectrometry of TC-ODN. **(D)** Representative images of cellular uptake of odanacatib and TC-ODN. **(E)** The fluorescence intensity of odanacatib and TC-ODN at each time point is presented (*n* = 3). **(F)** Representative images of gelatin spectrum test for CTSK enzyme activity and the semi quantitative analysis (*n* = 3). **(G)** Cytotoxicity of TC-ODN *in vitro* (*n* = 3). Data are presented as means ± SD. *p < 0.05 and ***p < 0.001 by one-way ANOVA.

TC–ODN was synthesized as shown in Figure 1A. According to the results of ^1^H-NMR, the hydrogens of the benzene ring of TC-ODN showed peaks at a and i, the hydrogens of the amino of TC-ODN showed peaks at j, the hydrogens of the methyl of TC-ODN showed peaks at b, c, and g, the hydrogens of the hydroxyl of TC-ODN showed peaks at e and f, the hydrogens of the methylene of TC-ODN showed peaks at d and h. (Figure 1B). Meanwhile, the results of MS showed that the molecular ion peak ([M + H]^+^) of TC-ODN was 1055 (Figure 1C). These findings confirmed that TC-ODN was successfully synthesized.

The results of cellular uptake test showed that TC-ODN could be absorbed into PDLSCs successfully (Figure 1D). Although the absorption rate of TC-ODN was lower than which of odanacatib at 6 and 12 h, there was no significant difference between TC-ODN and odanacatib at 24 and 48 h (Figure 1E). The results of the gelatin spectrum test showed that gelatin could be effectively degraded by CTSK, and the degradation process could be blocked by both odanacatib and TC-ODN. There was no significant difference between odanacatib and TC-ODN in inhibiting CTSK enzyme activity *in vitro* (Figure 1F).

To determine the security of TC-ODN *in vitro*, the cytotoxic effect of TC-ODN on PDLSCs was detected. As shown in Figure 1G, negligible cytotoxicity was displayed by TC-ODN at the concentrations of 1 μmol/L, while the cytotoxic effects were significantly displayed when the concentration of TC-ODN equaled to 10 μmol/L. So 1 μmol/L TC-ODN was chosen in the following experiments.

### Regulation of macrophage polarization by TC-ODN to inhibit local inflammation

**FIGURE 2:**
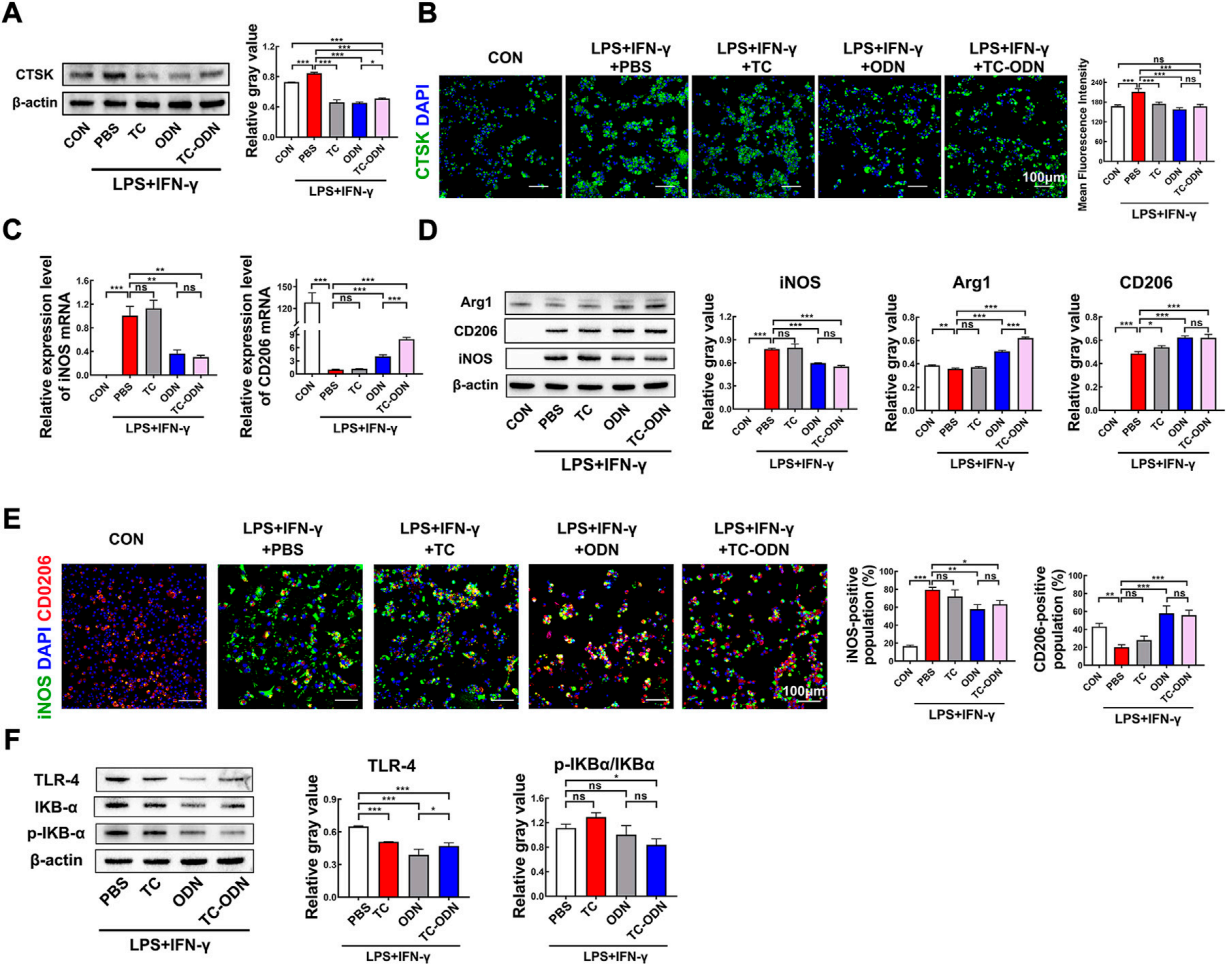
Regulation of macrophage polarization by TC-ODN to inhibit local inflammation. **(A)** Western blot analysis of the protein levels of CTSK in macrophages with different treatments and the semi-quantitative analysis in each group (*n* = 3). **(B)** Representative images of immunofluorescence staining to detect the expression of CTSK in macrophages and the mean fluorescence intensity (*n* = 3). **(C)** RT-PCR analysis of the expression of iNOS and CD206 in macrophages after different treatments (*n* = 3). **(D)** Western blot analysis of phenotype markers in macrophages under the inflammatory medium system after different treatments (*n* = 3). pro-inflammatory macrophage’s marker, iNOS; anti-inflammatory macrophage’s markers, CD206 and Arg1. **(E)** Representative fluorescence images of the macrophages phenotypes after different treatments (*n* = 3). **(F)** Western blot analysis of the protein levels of TLR-4, IKBα, and p-IKBα, after 30 min of incubation with the inflammatory medium system (*n* = 3). Data are presented as means ± SD. *p < 0.05, **p < 0.01 and ***p < 0.001 by one-way ANOVA.

Macrophage serves as a crucial cellular component of innate immunity involved in the pathogenesis of periodontitis, regulating polarization of macrophage has become a promising therapeutic approach for inflammatory diseases (Wang et al., 2019). LPS and interferon-γ (IFN-γ) were added to induce an inflammatory environment. Simultaneously, tetracycline, odanacatib, or TC-ODN (1 μmol/L) were added to the culture medium, respectively. After incubation for 24 h, the cells were harvested. Firstly, we verified the expression of CTSK by Western blot and immunofluorescence staining. Both results showed that the expression of CTSK was significantly up-regulated in an inflammatory environment, while odanacatib and TC-ODN could markedly reduce the level of CTSK expression. These results indicated that not only CTSK enzyme activity but its expression could be reduced by odanacatib or TC-ODN under inflammatory environment (Figures 2A,B).

Notably, we further assessed representative makers of pro-inflammatory and anti-inflammatory macrophages to explore the effect of TC-ODN on macrophage polarization. The results showed that the mRNA and protein level of iNOS (pro-inflammatory macrophage’s maker) significantly increased in an inflammatory environment, which was blunted by the administration of odanacatib and TC-ODN (Figures 2C,D). Meanwhile, expression of CD206 and Arg1 (anti-inflammatory macrophage’s maker) were markedly increased after administration of odanacatib and TC-ODN, and no significant difference was found between odanacatib and TC-ODN treatment (Figures 2C,D). Confocal microscopy was used to visualize the phenotype of macrophages, the results showed that treatment of odanacatib and TC-ODN decreased the pro-inflammatory subpopulation (iNOS-positive) and significantly increased the anti-inflammatory subpopulation (CD206-positive), which were consistent with the above (Figure 2E). Collectively, these results confirmed that TC-ODN could promote macrophage polarization from the pro-inflammatory phenotype to the anti-inflammatory phenotype under inflammatory environment.

Previous studies have demonstrated that the Toll-like receptor-4 (TLR-4)/NF-κB pathway plays an important role in the regulation of macrophage polarization (Porta et al., 2015; Akaishi and Abe, 2018; Li et al., 2018; Wang et al., 2019). TLR-4 and representative targets of the NF-κB pathway (IKBα and p-IKBα) were detected by Western blot. The results showed that treatment of odanacatib and TC-ODN decreased the protein expression level of TLR-4 and the ratio of p-IKBα/IKBα under inflammatory environment (Figure 2F). To sum up, these results indicated that TC-ODN could convert macrophages toward an anti-inflammatory phenotype *via* down-regulation of TLR-4/NF-κB signaling pathways in an inflammatory environment.

### Regulation of osteogenetic differentiation of PDLSCs by TC-ODN in an inflammatory environment to promote bone regeneration

**FIGURE 3:**
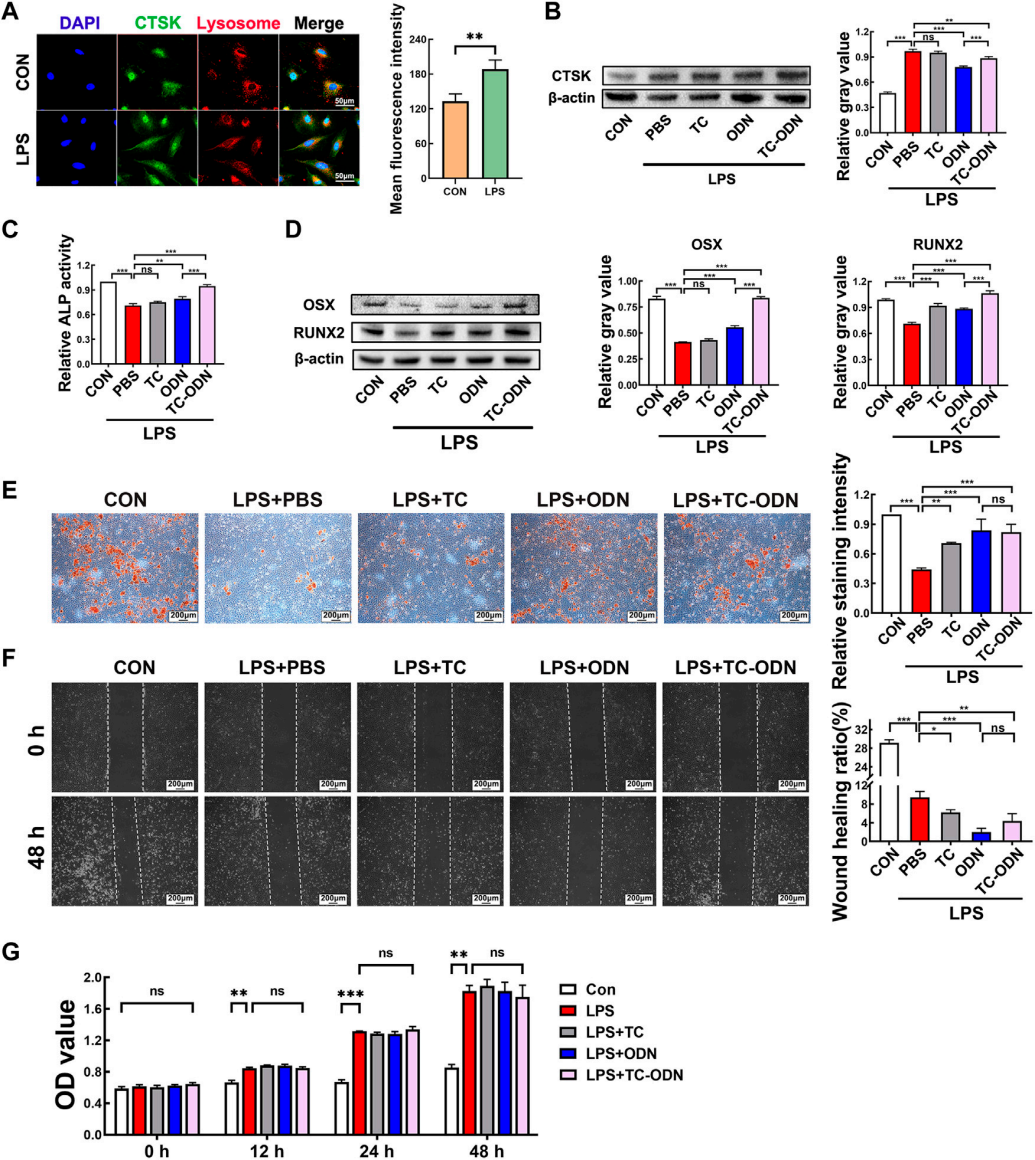
Regulation of osteogenetic differentiation of PDLSCs by TC-ODN in an inflammatory environment to promote bone regeneration. **(A)** Representative images of immunofluorescence staining to detect the expression of CTSK in PDLSCs and the mean fluorescence intensity (*n* = 3). **(B)** Western blot to detect the expression of CTSK in PDLSCs with different treatments and the semi-quantitative analysis in each group (*n* = 3). **(C)** ALP activity in each group (*n* = 3). **(D)** The expressions of Osterix and RUNX2 in different groups were detected by Western blot after 7 days of osteogentic differentiation (*n* = 3). **(E)** Representative images of alizarin red staining and the relative staining intensity (*n* = 3). **(F)** Representative images of the scratch wound assay and the wound healing ratio (*n* = 3). **(G)** Cell proliferation assayed by CCK-8 (*n* = 3). Data are presented as means ± SD. *p < 0.05, **p < 0.01 and ***p < 0.001 by one-way ANOVA **–**(B to I) and Student’s t-test **(A)**.

As periodontal regeneration is the most optimal treatment strategy for periodontitis, and PDLSCs play a quite important role in periodontal regeneration (Liang et al., 2021; Wu et al., 2021; Zhao et al., 2021), we explored the effect of TC-ODN on PDLSCs under inflammatory environment. Firstly, we confirmed the expression of CTSK in PDLSCs, which is not limited to lysosomes (Figure 3A). In addition, the expression of CTSK was also up-regulated in PDLSCs in an inflammatory environment (Figures 3A,B), and both TC-ODN and odanacatib could partially block the up-regulation of CTSK (Figure 3B).

We further assessed the effect of TC-ODN on the biological characteristics of PDLSCs in an inflammatory environment. The results showed that ALP activity and the expressions of Osterix and RUNX2 in PDLSCs were significantly decreased in the inflammatory group, which could be notably rescued by TC-ODN and odanacatib. What’s more, TC-ODN showed a stronger rescue ability than odanacatib (Figures 3C,D). Similar to the above results, the calcium nodule formation was suppressed in the inflammatory group, while TC-ODN or odanacatib-treated PDLSCs displayed an increased number of calcium nodules in an inflammatory environment (Figure 3E). In general, these results demonstrated that the osteogenetic capacity of PDLSCs was suppressed in an inflammatory environment. Notably, this suppressed osteogenetic capacity could be significantly rescued by TC-ODN.

In addition, the scratch wound assay showed that migration of PDLSCs was obviously inhibited in an inflammatory environment, and TC-ODN treatment could significantly aggravate this inhibitory effect (Figure 3F). On the other hand, the CCK-8 assay showed that cell proliferation was promoted in an inflammatory environment, which would not be changed by TC-ODN (Figure 3G).

### The bone-targeted capacity of TC-ODN *in vivo*

**FIGURE 4:**
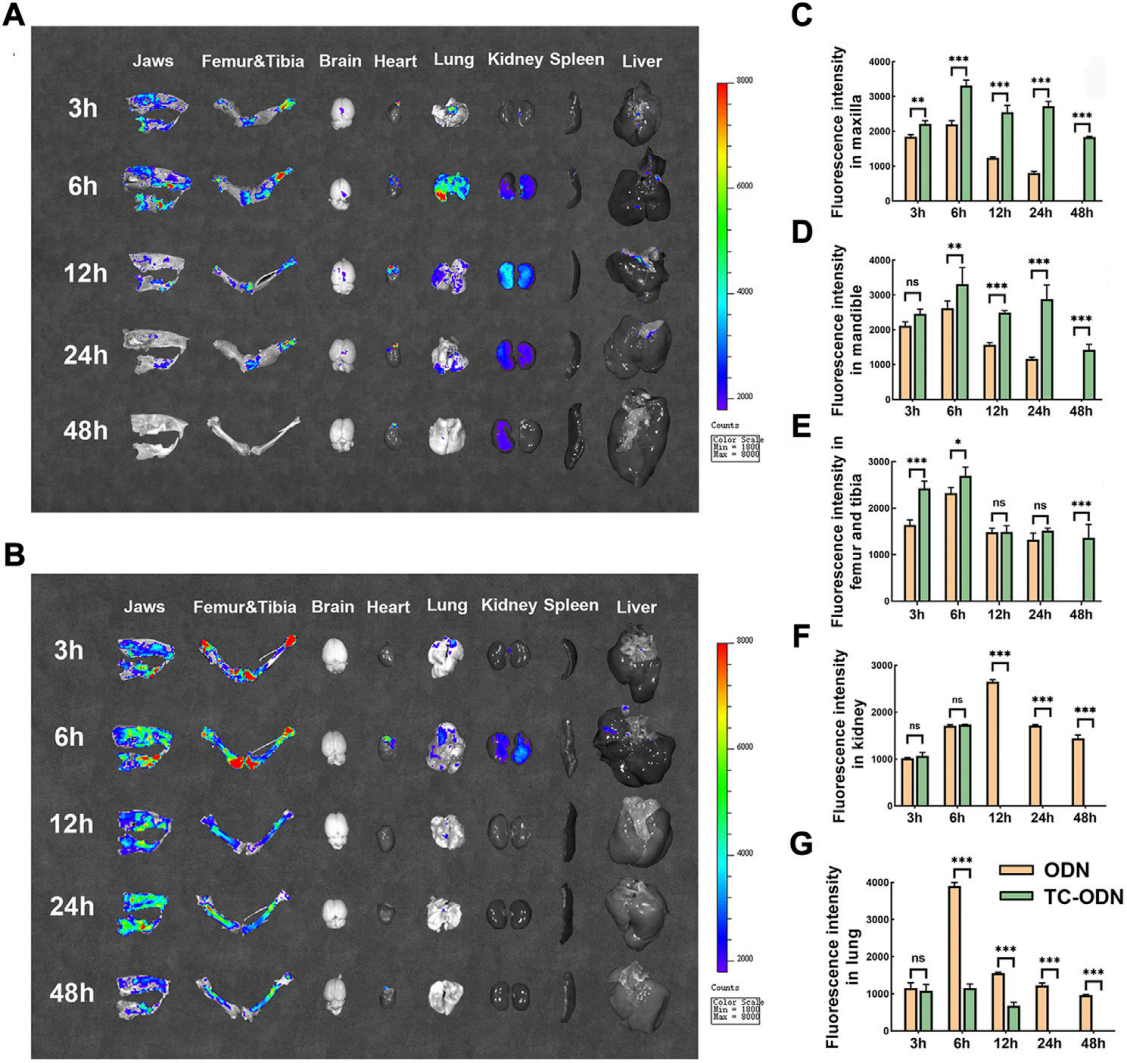
The bone-targeted capacity of TC-ODN in vivo. **(A)** Representative images of tissue distribution of odanacatib-FITC at each time point. **(B)** Representative images of tissue distribution of TC-ODN-FITC at each time point. **(C–G)** The fluorescence intensity of odanacatib and TC-ODN in maxilla, mandible, femur and tibia, kidney, and lung, respectively (*n* = 3). Data are presented as means ± SD. *p < 0.05, **p < 0.01 and ***p < 0.001 by one-way ANOVA.

To confirm the bone-targeted capacity of TC-ODN *in vivo*, the biodistribution of TC-ODN in rats was detected using an IVIS imaging system. The Organ imaging showed that in odanacatib group, the highest FITC expression was detected in the jaws, femur, tibia, lung, and kidney within 24 h. Modest expression was detected in the brain, heart, and liver, while no expression was detected in the spleen (Figure 4A). In TC-ODN group, the highest FITC expression in bone tissues lasted for 48 h, while the FITC expression could hardly be detected in the lung, kidney, and other organs after 6 h (Figure 4B). Further quantitative analysis of fluorescence intensity showed that compared with odanacatib group, the expression of FITC in the maxilla, mandible, femur, and tibia was higher in TC-ODN group at most times points (Figures 4C–E). Additionally, in TC-ODN group, the FITC fluorescence signal could not be detected in the kidney after 6 h and in the lung after 12 h (Figures 4F,G). These results demonstrated that this bone-targeted conjugate can be safely delivered to jaws and long bones without detectable side effects.

### Therapeutic efficacy of TC-ODN in ovariectomized osteoporosis

**FIGURE 5:**
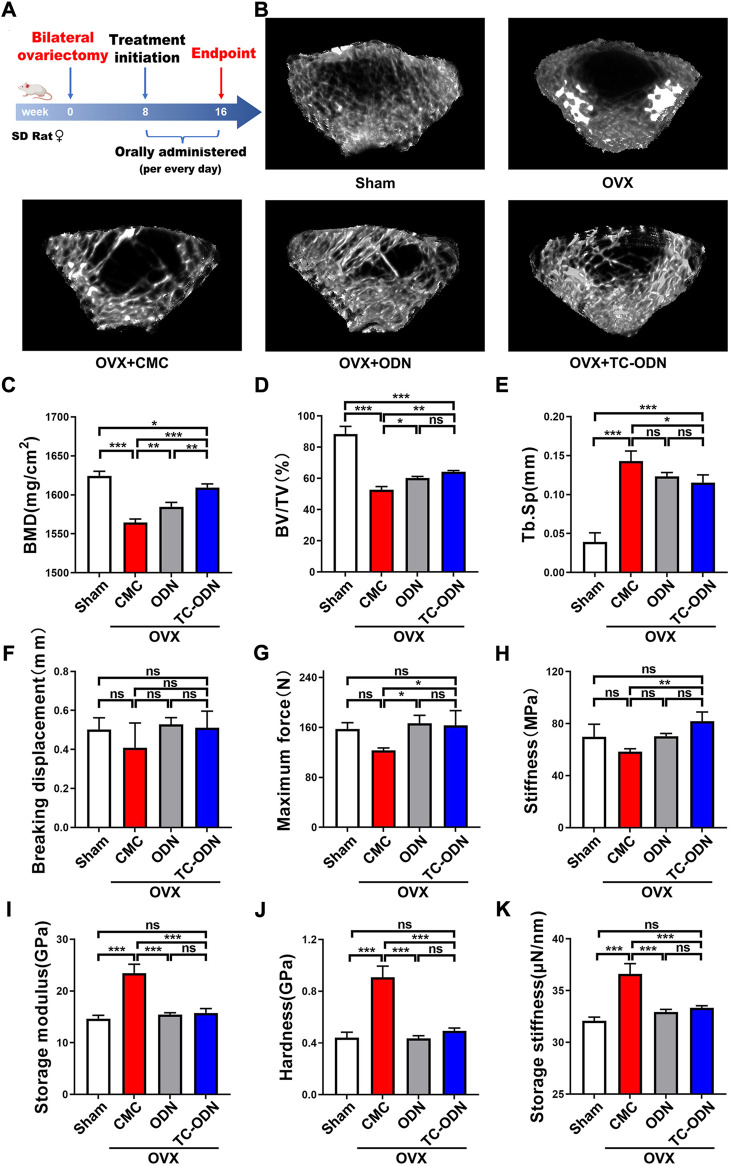
Therapeutic efficacy of TC-ODN in ovariectomized osteoporosis. **(A)** The therapeutic design of the osteoporosis rat. **(B)** Femoral assessed by Micro-CT at the endpoint in each group. Bone mineral density (BMD) **(C)**, ratio of bone volume to the total tissue volume (BV/TV) **(D)**, and trabecular separation (Tb. Sp) **(E)** in femoral in each group analyzed according to Micro-CT (*n* = 3). Breaking displacement **(F)**, maximum force **(G)**, and stiffness **(H)** in femur analyzed according to Three-point Bending test (*n* = 3). Mandibular cortex was used for test of storage moduli **(I)**, hardness **(J)**, and storage stiffness **(K)** (*n* = 3). Data are presented as means ± SD. *p < 0.05, **p < 0.01 and ***p < 0.001 by one-way ANOVA.

Odanacatib plays a quite important role in osteoporosis therapy by inhibiting CTSK (Drake et al., 2017). To evaluate the effect of systemic application of TC-ODN on promoting bone regeneration, an ovariectomized rat model was adopted. Femoral trabecular bone mass was assessed by Micro-CT (Figure 5B). As a result, the bone mineral density (BMD) in TC-ODN group was significantly higher than that in CMC and even odanacatib group (Figure 5C). Furthermore, bone volume fraction (BV/TV, %) and trabecular separation (Tb. Sp, mm) in TC-ODN group were similar to those in odanacatib group and significantly different from those in CMC group (Figures 5D,E). These results notably suggested that systemic application of TC-ODN could effectively increase BMD in osteoporosis rats.

The results of the three-point bending test showed that no significant difference could be found in breaking displacement among all groups (Figure 5F; Supplementary Table S2). Maximum force and stiffness in rats treated with TC-ODN were significantly higher than those in CMC group, similar to those in sham and odanacatib groups (Figures 5G,H; Supplementary Table S2). In addition, the results of the nanoindentation test showed that storage moduli, hardness, and storage stiffness in TC-ODN group were similar to those in odanacatib or sham group and significantly lower than those in CMC group (Figures 5I–K; Supplementary Table S2). TC-ODN could significantly increase BMD and improve the biomechanical properties, which indicated that TC-ODN could effectively decrease bone fragility and fracture risk in osteoporosis. Furthermore, TC-ODN is more effective than ODN in the treatment of osteoporosis.

### Therapeutic efficacy of TC-ODN in rat experimental periodontitis

**FIGURE 6:**
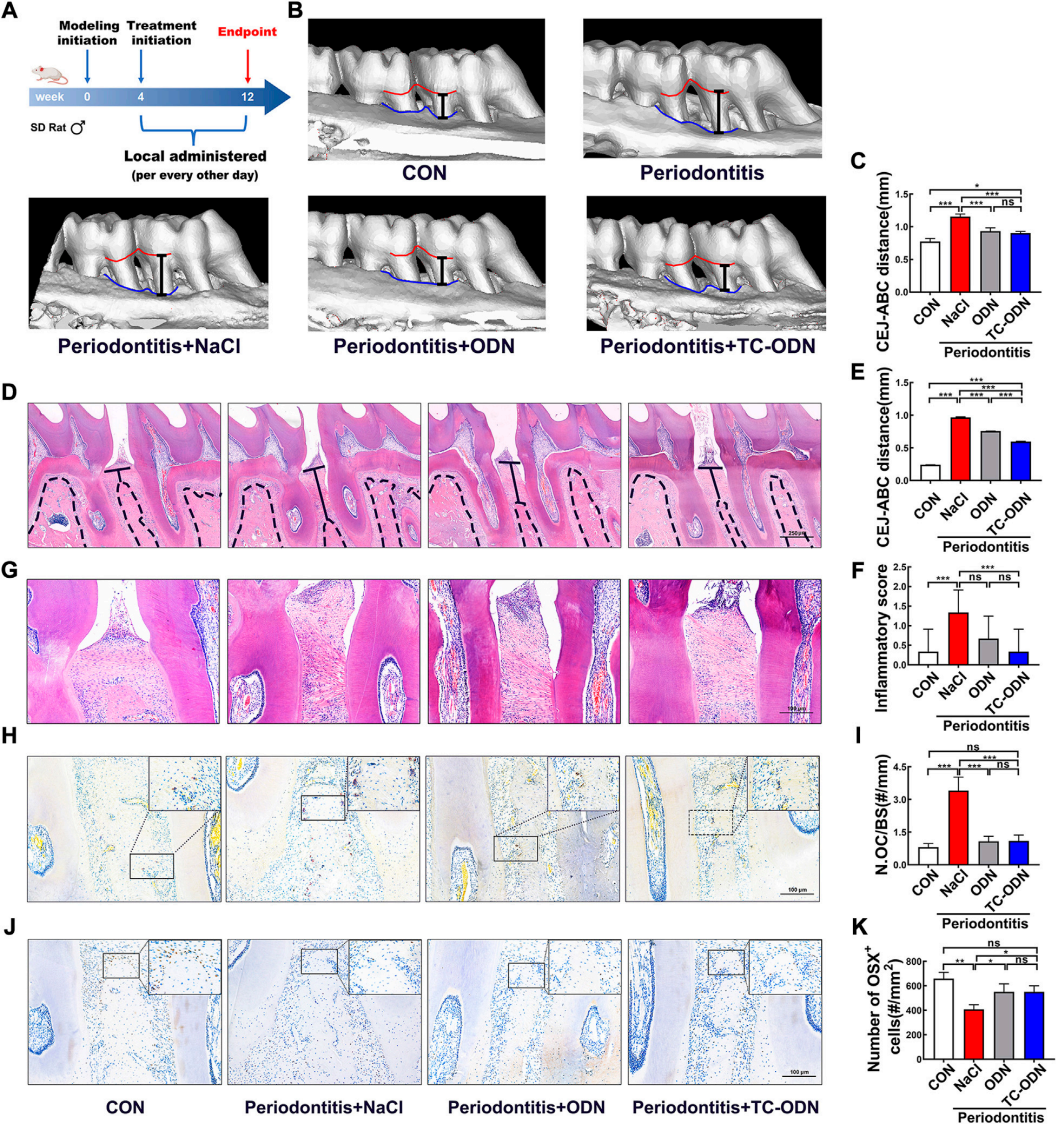
Therapeutic efficacy of TC-ODN in rat experimental periodontitis. **(A)** The therapeutic design of the experimental periodontitis. **(B)** Micro-CT scanning at the endpoint in each group. **(C)** The linear distance from cementoenamel junction to alveolar bone crest (CEJ-ABC) determined according to Micro-CT (*n* = 3). **(D)** Representative images of hematoxylin and eosin **(H,E)** staining of the teeth and periodontal tissues in each group. **(E)** The CEJ-ABC distance according to H&E staining (n = 3). **(F)** Representative images of H&E staining of the gingival tissue between the first molar and the second molar in each group. **(G)** Score of inflammatory cells according to H&E staining (*n* = 3). **(H)** Representative images of TRAP staining between the roots of the first molar and the second molar. **(I)** TRAP-positive osteoclasts were counted (*n* = 3). **(J)** Representative images of IHC staining of Osterix between the roots of the first molar and the second molar. **(K)** Number of Osterix^+^ cells (*n* = 3). Data are presented as means ± SD. *p < 0.05, **p < 0.01 and ***p < 0.001 by one-way ANOVA.

Inhibiting CTSK could prevent inflammation and bone erosion caused by periodontitis (Hao et al., 2015a; Hao et al., 2015b; Chen et al., 2016), but it remains unclear whether the damaged alveolar bone caused by periodontitis can be regenerated by CTSK. The linear distance from the cementoenamel junction to the alveolar bone crest (CEJ-ABC) was determined by both Micro-CT (Figures 6B,C) and H&E staining (Figures 6D,E). As presented in Figures 6C–E, the CEJ-ABC distances in TC-ODN and odanacatib groups were significantly shorter than that in periodontitis group. Interestingly, the results of H&E staining showed that the therapeutic effect of TC-ODN was significantly better than that of odanacatib (Figure 6E).

To explore the impacts of TC-ODN on inflammatory cells, osteoclasts, and osteoblasts, H&E staining, TRAP staining, as well as IHC staining of Osterix were performed. The results showed that the periodontitis group had the highest score of inflammatory cells (Figures 6F,G), the largest number of osteoclasts (Figures 6H,I), and the fewest osteoblasts (Figures 6J,K) among all the groups. Notably, TC-ODN could not only significantly reduce the inflammatory score and the number of osteoclasts but also raise the number of osteoblasts, and TC-ODN exhibited a better effect than odanacatib in inhibiting inflammatory cell infiltration. These results demonstrated that TC-ODN could ameliorate the local immune microenvironment, inhibit the alveolar bone resorption process as well as promote bone regeneration in periodontitis simultaneously, which ultimately realized the process of alveolar bone regeneration under the periodontitis environment.

## Discussion

The present study constructed a bone-binding multifunctional drug (TC-ODN), and we revealed that TC-ODN could promote macrophage polarization from pro-inflammatory phenotype to anti-inflammatory phenotype and enhance osteogenic differentiation of PDLSCs in an inflammatory environment. Notably, we verified this conjugate achieved odanacatib targeting delivery to the bone to reduce adverse effects. *In vivo* study, we demonstrated that systemic application of TC-ODN could increase BMD in osteoporosis, and for the first time we found that periodontal application of TC-ODN could promote the bone regeneration of damaged alveolar bone in existing periodontitis.

As a broad-spectrum antibiotic, tetracycline has been widely used in the local treatment of periodontitis (Schwach-Abdellaoui et al., 2001; Aimetti et al., 2004; Mundargi et al., 2007; Wei et al., 2021). However, tetracycline is not correlated with bone healing promotion, and administration of tetracycline alone cannot achieve the functional regeneration of periodontal tissues in periodontitis. In our previous study, deficiency of CTSK could promote alveolar bone regeneration after tooth extraction in mouse, which cannot be achieved by tetracyclines (Zhang et al., 2021). What’s more, recent literature has confirmed that inhibition of CTSK could make the local immune microenvironment viable, and suppress the osteoclast-mediated bone resorption process (Chen et al., 2016). The above results indicated that inhibiting CTSK may be a promising strategy to promote bone healing in the treatment of periodontitis. Odanacatib is a selective inhibitor of CTSK which is served as the most promising drug for osteoporosis, it also effectively prevents the occurrence of periodontitis. Although odanacatib achieved remarkable advances, it was discontinued because of the increased risk of cerebrovascular accidents (Mullard, 2016; McClung et al., 2019; Stone et al., 2019; Binkley et al., 2021). Coincidentally, as an anti-microbial agent for treating periodontitis, tetracycline could serve as a good candidate for constructing a bone-targeted delivery system of drugs (Lee et al., 2016; Xie et al., 2018). To reduce the side effects of odanacatib in non-bone tissues, a bone-binding multifunctional drug was constructed in this study by conjugation of tetracycline with odanacatib.

*In vitro* study, we found the protein expression level of CTSK was decreased in macrophages and PDLSCs after treatment with TC-ODN under inflammatory environment, and this effect was also found in breast cancer cells (Vashum et al., 2021). These results suggested that TC-ODN could exert a therapeutic effect by inhibiting CTSK activity and expression. For the first time, we found that TC-ODN could promote macrophages polarizing from pro-inflammatory phenotype to anti-inflammatory phenotype in an inflammatory environment by regulating the TLR-4/NF-κB pathway. The pro-inflammatory cytokines secreted by pro-inflammatory subpopulations were closely related to osteoclastogenesis (Lam et al., 2000; Hao et al., 2015a; Hao et al., 2015b), which was consistent with the findings that the number of osteoclasts was significantly decreased after treatment with TC-ODN in periodontitis rats. Our previous study demonstrated that deletion of CTSK could promote jaw bone marrow mesenchymal stem cell proliferation and differentiation in a normal microenvironment (Zhang et al., 2021). In this study, we found this multifunctional CTSK inhibitor could rescue the osteogenetic capacity of PDLSCs in an inflammatory environment. Therefore, this multifunctional drug is a promising agent for promoting damaged alveolar bone regeneration in existing periodontitis.

Periodontitis and osteoporosis have been important public health concerns, and there are many hypotheses about the link between these two resorptive bone diseases (Curtis et al., 2017; He et al., 2018; Makras et al., 2020; Wei et al., 2021). On the one hand, previous literature indicated that the reduction of BMD in osteoporosis was correlated with the effect of oral bacterial biofilm, which can accelerate alveolar bone resorption, and then lead to the rapid development of periodontal disease (Xu et al., 2021). Meanwhile, systemic inflammatory mediators which promoted osteoclast production led to the change in local tissue response, which caused alveolar bone loss (Hong et al., 2021; Xu et al., 2021). On the other hand, as a chronic inflammatory disease, periodontitis is mainly caused by bacterial plaque biofilms, and local periodontal tissue usually has an immune response that increased the production of cytokines associated with periodontal disease, which may accelerate systemic bone resorption (Hong et al., 2021; Xu et al., 2021). So periodontitis may be also a risk factor affecting systemic bone resorption, particularly for postmenopausal women (Mashalkar et al., 2018; Hong et al., 2021). So far, the association between these two resorptive bone diseases is still controversial. However, these two diseases share common characteristics, so we are committed to constructing a multifunctional drug to treat both diseases. Importantly, the multifunctional drug has shown an excellent therapeutic effect on osteoporosis and periodontitis due to its antimicrobial, anti-inflammatory, and bone healing promotive properties, and we will establish an appropriate delivery system of this multifunctional drug to treat these two diseases simultaneously in further study.

*In vivo* study, TC-ODN achieved the enrichment of drugs in long bones and jaws, it is the reason why the therapeutic effect on BMD was significantly better in osteoporosis rats compared with odanacatib. Meanwhile, we found both TC-ODN and ODN could decrease the inflammatory score in periodontitis rats, which was consistent with the immunoregulatory effect of CTSK (Hao et al., 2015a; Hao et al., 2015b; Chen et al., 2016). Notably, as we expected, TC-ODN was significantly better than ODN in inhibiting local inflammation due to the properties of tetracycline.

Although TC-ODN has an excellent therapeutic effect on these two public health problems, there are still many challenges to surmount from laboratory to bedside. For further translation to clinical application, it is valuable to study the delivery system of TC-ODN. For the treatment of periodontitis, it is of great necessity to use local drug delivery systems (i.e., microspheres, nanosystems, and gels) (Wei et al., 2021). On the contrary, as systemic bone disease, osteoporosis is usually treated by systemic administration (Black and Rosen, 2016). Therefore, various designs of drug delivery systems are required to satisfy different clinical requests. Although more work needs to be done before clinical application, this multifunctional drug heralds a new era in managing these two diseases simultaneously.

## Conclusion

We constructed a bone-binding multifunctional drug and proved its safety, effectiveness and bone targeting ability. We confirmed that systematic application of this multifunctional drug could effectively increase the BMD of osteoporosis rats and improve the biomechanical strength of bone. Notably, periodontal application of this drug could also promote the damaged alveolar bone regeneration in the existing periodontitis by promoting macrophages polarization towards anti-inflammatory phenotype and rescuing the osteogenetic capacity of PDLSCs. Hence, we believe this bone-binding multifunctional drug suggests a new method for the treatment of periodontitis and osteoporosis.

## Data Availability

The original contributions presented in the study are included in the article/Supplementary Material, further inquiries can be directed to the corresponding authors.
