# Supplementary material for: A multifunctional drug consisting of tetracycline conjugated with odanacatib for efficient periodontitis therapy
Source: Front Pharmacol. 2022 Oct 26;13:1046451. doi: 10.3389/fphar.2022.1046451 (PMC9643870; doi:10.3389/fphar.2022.1046451)
Supplement: Supplementary file 1 [file Table1.docx]

**Supplementary Materials**

Table S1 Primer sequence used for quantitative RT-PCR

| Gene | Primer sequence | Product size |
| --- | --- | --- |
| *iNOS* | F: CAGGCTTGGGTCTTGTTAGC  R:TTGTTGGGCTGGGAATAGCA | 200 |
| *CD206* | F: ACAGACGGACGAGGAGTTCA  R: ATAGGCACAGAAGGGTCGGT | 200 |
| *Actin* | F: CCCATCTATGAGGGTTACGC  R: TTTAATGTCACGCACGATTTC | 150 |

**Table S2 Three-point bending test and nanoindentation test**

|  | Sham | OVX+CMC | OVX+odanacatib | OVX+TC-ODN |
| --- | --- | --- | --- | --- |
| **Three-point Bending test** | |  |  |  |
| Breaking  displacement (mm) | 0.502  ±0.049 | 0.409  ±0.103 | 0.529  ±0.028 | 0.511  ±0.069 |
| Maximum force (N) | 157.5  ±8.2 | 123.3  ±3.1 | 166.7  ±10.5 | 163.3  ±19.3 |
| Stiffness (GPa) | 69.8  ±7.9 | 58.4  ±1.8 | 70.4  ±1.7 | 82.0  ±5.7 |
| **Nanoindentation test** | |  |  |  |
| Storage moduli  (GPa) | 14.66  ±0.65 | 23.45  ±1.74 | 15.46  ±0.34 | 15.75  ±0.86 |
| Hardness (GPa) | 0.443  ±0.040 | 0.907  ±0.088 | 0.435  ±0.022 | 0.494  ±0.021 |
| Storage Stiffness  (μN nm^-1^) | 32.09  ±0.35 | 36.60  ±1.00 | 32.95  ±0.24 | 33.34  ±0.21 |
